# Prospective 100 first results of immediate scoliosis correction with the new lyon brace: ARTbrace

**DOI:** 10.1186/1748-7161-9-S1-O35

**Published:** 2014-12-04

**Authors:** Jean Claude de Mauroy, Sophie Pourret

**Affiliations:** 1 Clinique du Parc, Lyon, France; 2 Lecante, Lyon, France

## Background

The immediate reducibility of the Cobb angle in brace is the fundamental parameter of success of non-surgical orthopedic treatment of scoliosis.

## Aim

The objective of this work is to present the first results of the new Lyon brace (Asymmetrical Rigid Torsion brace) with immediate realization of the brace without plaster cast.

## Design

The new Lyon brace is constructed with two asymmetrical lateral polycarbonate pieces connected posteriorly at the midline by a vertical incurved bar. The brace reproduce a twisted column in the opposite direction of scoliosis. The shape is obtained by superposition of three segmental electronic moldings which will be superimposed by a new software: OrtenShape.

## Results

The results of a prospective series of the first 100 consecutive patients were studied using EOS X-ray and compared with results obtained by other braces.

Radiologically, in the frontal plane, the immediate in brace reduction is on average (0.72) (0.63 +- 0.19 for thoracic curves & 0.76 +- 0.26 for lumbar curves. These results can be classified:

- Depending on the type of curvature:

thoracolumbar (0.98),

lumbar (0.71),

double major (0.67)

lumbar (0.67),

thoracic (0.64)

- According to the criteria of the SRS (40 cases):

thoracic curves (0.66),

lumbar (0.83)

- According to the initial angulation:

20 -29° = (0.80)

30-39° = (0.65)

> 40° = (0.45)

In 51 cases with initial kyphosis <30°, improving the flat back is 9.25° from 19° to 28.25°.

Clinically, the push-up effect is : 1.64 cm.

After at least 1 month of continuous wearing, for thoracic rib hump improvement is (0.50) and for lumbar (0.85).

The improvement is 40% compared to the old plaster cast and Lyon brace and 60% compared to the Chêneau-Münster brace.

All radiological and clinical parameters improved significantly.

## Conclusion

The new Lyon brace (ARTbrace) allows a better immediate in-brace reduction than the old Lyon brace. without need for a preliminary plaster cast.

